# Electromagnetic Interference Shielding Performances of Carbon-Fiber-Reinforced PA11/PLA Composites in the X-Band Frequency Range

**DOI:** 10.1021/acsomega.3c01656

**Published:** 2023-06-15

**Authors:** Bedriye Ucpinar Durmaz, Alp Oral Salman, Ayse Aytac

**Affiliations:** † Department of Chemical Engineering, Engineering Faculty, Kocaeli University, Kocaeli 41380, Türkiye; ‡ Department of Electronics and Communication Engineering, Engineering Faculty, Kocaeli University, Kocaeli 41001, Türkiye; § Polymer Science and Technology Programme, Kocaeli University, Kocaeli 41001, Türkiye

## Abstract

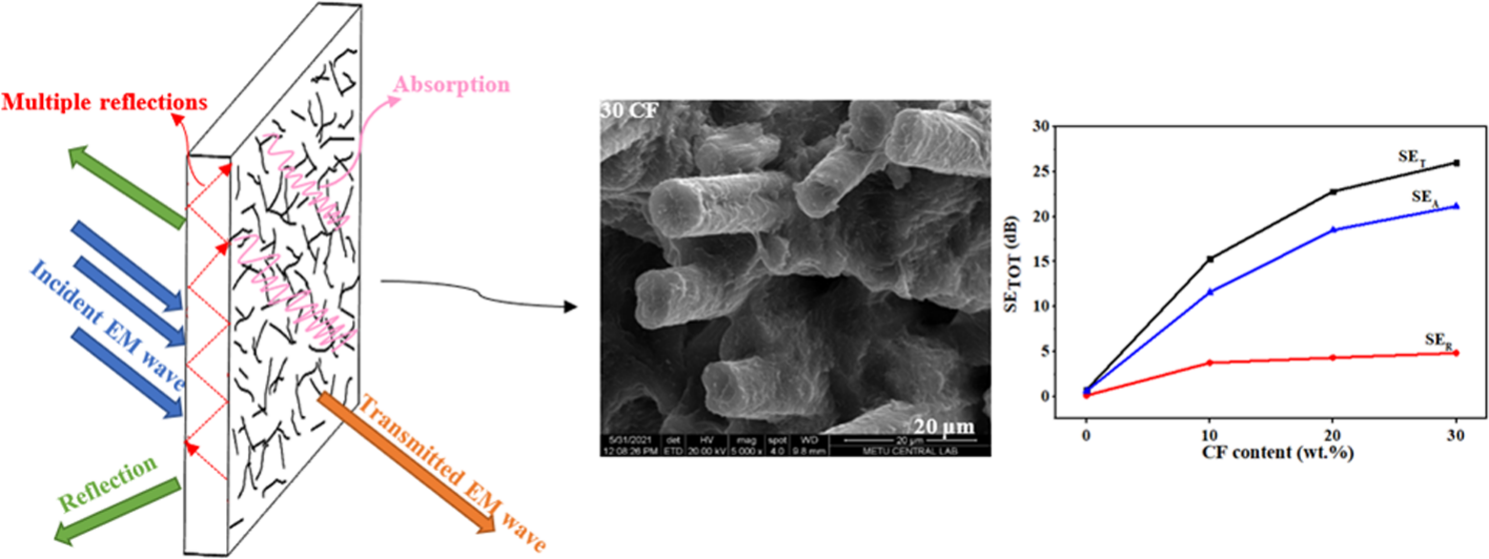

To solve the problem of increasing electromagnetic pollution, it is crucial to develop electromagnetic interference (EMI) shielding materials. Using lightweight, inexpensive polymeric composites instead of currently used metal shielding materials is promising. Therefore, bio-based polyamide 11/poly(lactic acid) composites with various carbon fiber (CF) amounts were prepared using commercial extrusion and injection/compression molding methods. The prepared composites’ morphological, thermal, electrical conductivity, dielectric, and EMI shielding characteristics were investigated. The strong adhesion between the matrix and CF is confirmed by scanning electron microscopy. The addition of CF led to an increase in thermal stability. As CFs formed a conductive network in the matrix, direct current (DC) and alternative current (AC) conductivities of the matrix increased. Dielectric spectroscopy measurements showed an increase in the dielectric permittivity/energy-storage capability of the composites. Thus, the EMI shielding effectiveness (EMI SE) has also increased with the inclusion of CF. The EMI SE of the matrix increased to 15, 23, and 28 dB, respectively, with the addition of 10–20–30 wt % CF at 10 GHz, and these values are comparable or higher than other CF-reinforced polymer composites. Further analysis revealed that shielding was primarily accomplished by the reflection mechanism similar to the literature data. As a result, an EMI shielding material has been developed that can be used in commercially practical applications in the X-band region.

## 1 Introduction

Rapid development of applications like electronics, telecommunications, radar, and so forth causes excessive electromagnetic (EM) waves.^1−3^ The EM noises create electromagnetic pollution, which is called electromagnetic interference (EMI). The performance of surrounding electronic devices, information communication security, and human health are seriously affected by this EMI.^4−6^ So, EMI is a serious problem in many industries, including communications, medical instruments, buildings like hospitals, military applications, and broadcasting. Therefore, the development of efficient, lightweight, and economical EMI shielding materials is important and attracts the attention of researchers and scientists.^4,7,8^ Various magnetic and electrically conductive materials are used in EMI shielding like metallic and polymeric composites and adhesives.^9−11^

EMI shielding can occur via three mechanisms. The EM waves may be reflected from the front surface of the shielding material, absorbed by the shielding material, or reflected on its internal surfaces. With the contribution of all of these mechanisms, the total EMI shielding effectiveness (EMI SE) of the shielding material is obtained.^12,13^ The EMI SE depends on several factors such as electrical conductivity, permittivity, permeability, and thickness. One essential factor for high EMI SE is electrical conductivity.^7,10,12^ Metals have long been employed as shields due to their high electrical conductivity (10^4^–10^5^ S/cm).^11^ However, they have shortcomings including high density, large size, limited flexibility, poor resistance to corrosion, and expensive and challenging processing.^4,13,14^ In this context, electrically conductive polymeric composites carry the potential as an alternative to metal-based shielding materials. The usage of these materials in EMI shielding applications is expanding due to their lightness, lower price, simple processing, and environmental stability.^5,13,15−17^ As EMI shielding materials, a number of polymeric matrices were investigated, including polyaniline,^11^ poly(phenylene sulfide),^18^ polyimide,^19^ polypropylene (PP),^5,15,17^ polycarbonate (PC),^20^ polyamide,^21,22^ and poly(lactic acid) (PLA).^15,16,23^ Several types of fillers, including silver,^18^ carbon nanotube,^15,20^ graphene/graphite,^16,22^ and carbon fibers,^17,19,21,24^ were incorporated into various matrices in order to create a conductive polymer composite. Among these fillers, metal particle–polymer composites often suffer from poor mechanical properties. Additionally, despite the fact that carbon nanotubes (CNTs) show potential in terms of conductivity and shielding, their expensiveness prevents their acceptance in the industrial environment.^25^ In this regard, high aspect ratio carbon fibers (CFs) have attracted attention to use in EMI shielding applications due to their large surface areas, lightness, high mechanical strength, and excellent electrical and thermal conductivity.^5,7,13,17,24,26−28^ A superior balance between mechanical and electrical properties has reportedly been found in CF-reinforced polymer composites compared to metal-reinforced ones.^25^ Moreover, CFs are regarded as a less expensive alternative to CNTs with similar characteristics.^5^ CF-reinforced polymer composites can be obtained using both continuous and discontinuous short CFs. Short CF-reinforced composites offer design flexibility since they may be made with an established, affordable manufacturing technique like injection molding.^25^ Meanwhile, it is known from our previous work that CF reinforcement significantly improves the mechanical properties and load-bearing capacity of a polymer matrix.^29^ Consequently, the usage areas of CF-reinforced polymer composites can be extended from high-performance engineering applications (automotive, aerospace) to EMI shielding. Numerous studies have examined the effect of CF addition on the EMI SE of polymers. SE measurements of nylon 6.6/CF composites produced by extrusion and injection molding were carried out by Keith et al.^21^ The findings demonstrated that SE gradually increased with the increase of CF. In a different study, Kaushal and Singh^17^ examined the electrical conductivity and EMI SE of PP/CF composites. The results demonstrated that 20 wt % CF loading provided 3.7 × 10^–3^ S/cm electrical conductivity and −32 dB EMI SE in the X-band region. The mechanical properties, electrical conductivity, and EMI SE values of polymer composites depend on the loading amount, size and shape, orientation, and dispersion of the filler. After a certain loading amount, there is no significant change in the properties, but on the contrary, there may be a decrease in the properties due to agglomeration of the filler.^24,30^ At high amounts of CFs, fibers agglomerate and become difficult to disperse, which can obstruct the creation of a conductive network and the shielding of EM waves throughout the matrix.^24^ Therefore, in this study, the highest CF loading amount was chosen as 30 wt %.

On the other hand, the majority of commercially used polymers are synthetic, for example, polyethylene (PE), polycarbonate (PC), polystyrene, polypropylene (PP), and polyamide 6, and their waste creates environmental pollution. As in many sectors, the rapid growth and short life cycles of materials in various industries lead to the formation of a large amount of ‘electronic waste’ (e-waste) both during fabrication and at end of life.^31^ By manufacturing electronic goods with bio-based and environmentally friendly polymers, these e-waste damages can be avoided. In this regard, studies on the production of EMI shielding materials from biopolymers as a matrix have increased in the last few years. Nath et al.^23^ produced PLA/thermoplastic polyurethane/carbon black biodegradable nanocomposites by a solution mixing method. They achieved EMI SE −27 dB in the X-band for 30 wt % carbon black addition. A study on PLA/poly(ethylene glycol)/MWCNT biodegradable nanocomposites was also conducted by Ahmad et al.^32^ The findings showed that PLA/PEG/MWCNT nanocomposites with 4 wt % MWCNTs exhibited EMI SE of 42 dB. As is evident, PLA is frequently investigated in these applications due to its biodegradability. PLA is produced from renewable sources, and it is biodegradable. Owing to its high modulus and tensile strength, it can also be utilized as a substitute for PE and PP. However, its shortcomings, including brittleness, slow rate of crystallization, and poor thermal stability, restrict its range of uses.^29,32^ As far as known, blending PLA with bio-based polyamide 11 (PA11) is a good solution to overcome these disadvantages. PA11 is a renewable bio-based polymer derived from castor oil. It has high flexibility, high impact, and moderate tensile strength, and is light. Additionally, it causes less environmental harm than synthetic polymers.^33^ In our earlier research, we reported that adding PA11 to PLA increased its flexibility, impact strength, and thermal stability. Moreover, tensile strength and Young’s modulus of PA11 improved by blending.^29,34^ In a different investigation, we added CF to the 60/40 (wt/wt) PA11/PLA blend that has the desired qualities, to explore the thermo-mechanical, mechanical, morphological, thermal, and rheological features. The findings demonstrated that CF greatly improved the tensile strength and Young’s and storage moduli of the PA11/PLA matrix.^29^ This investigation led to the hypothesis that high-performance PA11/PLA/CF composites would find use in a wider range of applications.

Therefore, it was decided that PA11/PLA/CF should be investigated further. CF-reinforced PA11/PLA composites were manufactured by extrusion and injection molding/compression molding processes. Because of their advantages over alternative processes, extrusion and injection/compression molding are widely used in commercial manufacturing. While extrusion offers benefits including ease of use, rapid production, versatility, and low cost, injection and compression molding also enable the creation of complex-shaped parts in large quantities with precise tolerances.^12,25^

The electrical, EMI SE, and thermal stability properties of PA11/PLA/CF composites were studied. Additionally, polymeric materials’ fire resistance needs to be increased to minimize the risk of a fire. Therefore, UL-94 vertical burning and limiting oxygen index (LOI) tests were performed to determine the flammability characteristics of the CF-reinforced composites. The impact of CF amounts (10–20–30 wt %) on the characteristics of PA11/PLA composites was also investigated.

## 2 Experimental Details

### 2.1 Materials

Polymers PA11 and PLA were used as matrix material. PA11 (Arkema, Rilsan-BESNO P40 TL) was obtained from Gültekin Plastik Profil San. Ve Tic. Ltd. Sti. Istanbul, Turkey. The renewable carbon ratio of PA11 is >89%. The density and melting temperature of PA11 are 1.04 g/cm^3^ and 181 °C, respectively. The PLA was purchased from NatureWorks with the trade name of Ingeo 2003 D. The density and melting temperature of PLA are 1.24 g/cm^3^ and 155 °C, respectively. PA-sized chopped CF with a bulk density of 575 g/L was acquired from DowAksa, Turkey.

### 2.2 Processing

The PA11/PLA/CF samples were manufactured utilizing a melt compounding procedure with an Xplore 15 mL co-rotating twin-screw extruder in a nitrogen atmosphere. Before the extrusion process, granules were vacuum-dried in an oven at 80 °C for 12 h. The compounding parameters were barrel temperature 210 °C, screw speed 75 rpm, and 3 min residence time. During the extrusion, polymer granules were fed together into a barrel and premixed for 2.15 min. Then, CF was fed and mixed for another 45 s. A blend of 60/40 (wt/wt) PA11/PLA was used as a matrix, and 10–20–30 wt % CF was added to this matrix. The extrudates were molded by using a mini-injection molder for obtaining ISO 527-2 5A tensile test specimens. The injection pressure was 10 bars, while the mold and melt temperatures were 30 and 210 °C, respectively. For EMI shielding characterization, specimens were formed by a compression molding machine. The molding was carried out under 60 bars, 220 °C, and 10 min. The sizes of the specimens were 10 × 10 × 0.1 cm^3^, respectively.

### 2.3 Characterization Tools

A scanning electron microscope (SEM, QUANTA 400 F) was used to explore the microstructure of the composites. Samples broken in liquid nitrogen were coated with gold before SEM examination to prevent arching.

The thermal stability of the PA11/PLA/CF composite was characterized by thermogravimetric analysis (Mettler Toledo, TGA 1). Under N_2_, 5–10 mg weight samples were heated at a rate of 10 °C/min from 25 to 600 °C. At the end of the analysis, the temperatures at which 5 wt % (*T*_d5_), 50 wt % (*T*_d50_), and maximum (*T*_max_) degradation of the samples’ weight loss occurs, and residual weight were determined.

LOI values were measured with Mares Analyzer (M-LOI-01) according to TS 11162-2 standard. Vertical burning test UL-94 was also performed to determine the flammability properties of composites according to ASTM D3801. Five samples of each composite were tested, and average values were reported.

The volume resistivity of the matrix and composites was determined by considering the ASTM D257 standard. The measurements were conducted by using a Keithley 6517B electrometer and a Keithley 8009 resistivity chamber. Five measurements were performed on 10 × 10 cm^2^ samples with a 1 mm average thickness and the average values were reported.

Dielectric spectroscopy was conducted in the range of 10^–2^ and 10^7^ Hz by using a Novocontrol α-A broad-band dielectric spectrometer at room temperature. For dielectric measurements, 2 × 2 cm^2^ and 1 mm thick samples were covered with a conductive silver paste to form electrodes. The real (ε_r_′) and imaginary (ε_r_″) parts of relative dielectric permittivity and AC conductivities of the composites were determined as a function of frequency.

**Figure 1:**
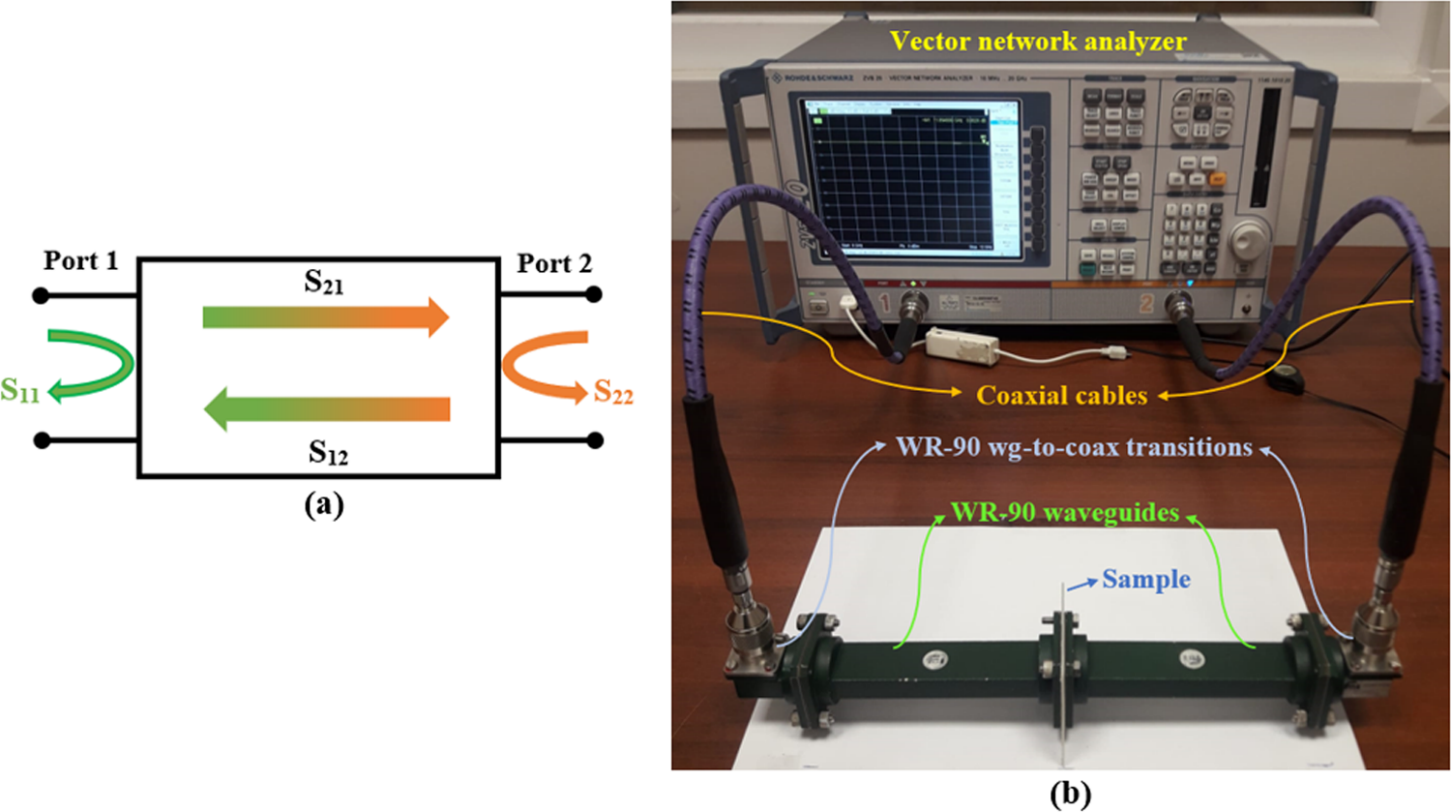
(a) Schematic representation of scattering parameters and (b) EMI SE measurement setup.

The EMI shielding performances of the samples in the range of 8.2–12.4 GHz were determined by using a two-port vector network analyzer (Rohde & Schwarz ZVB20 VNA) with a WR-90 waveguide setup (Figure 1). Full two-port calibration was applied from the end of the coaxial cables before the measurements on the VNA. The type of calibration was TOSM (true, open, short, match). For the measurement, the compression-molded samples were used with a size of 10 × 10 × 0.1 cm^3^. Scattering parameters (*S*-parameters) of *S*_11_ (input reflection of port 1) and *S*_21_ (transmission of port 1 to port 2) were obtained from the VNA to calculate the EMI SE and the representation of *S*-parameters is shown in Figure 1a.

Total EMI SE (SE_TOT_) includes the SE resulting from the absorption of EM power (SE_A_), the reflection from the material (SE_R_), and the multiple internal reflections (SE_M_). EMI SE of a material can be depicted as follows^19,32^
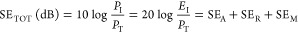
1*P*_T_ and *P*_I_ show the transmitted and incident powers of EM waves, respectively. Multiple reflections can be neglected where the thick shield and therefore SE_A_ are greater than 10 dB.^35^

The *S*-parameters were used for the calculation of the power coefficients of absorptivity (*A*), reflectivity (*R*), and transmittivity (*T*) via eq 2(22,36,37)
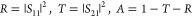
2The contributions of reflection and absorption mechanisms to the EMI SE_TOT_ were calculated by using *R*, *T*, and *A* values obtained through the following equations:^22,38^
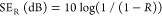
3
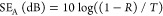
4
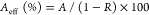
5

## 3 Results and Discussion

### 3.1 Morphology of the Samples

**Figure 2:**
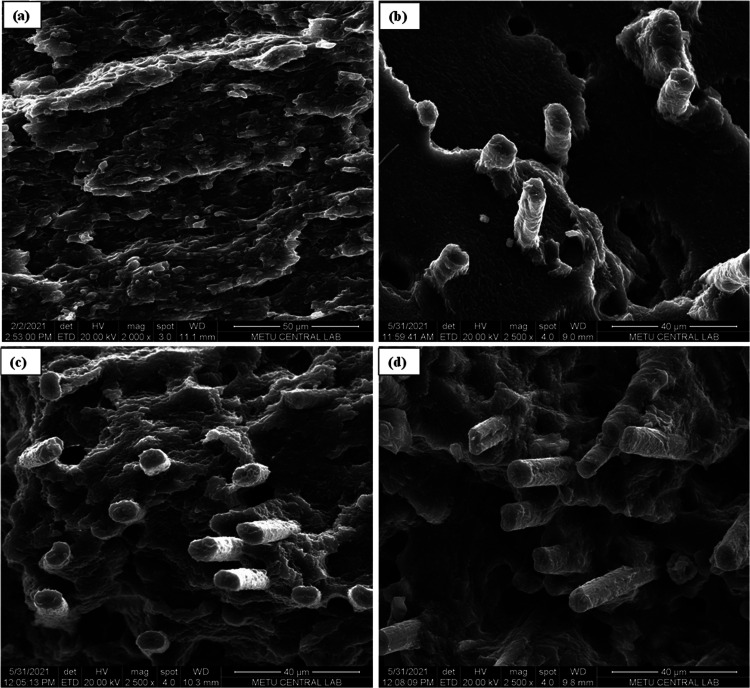
Cryo-fractured surface images of (a) PA11/PLA (×2000), and (b) 10 CF, (c) 20 CF, and (d) 30 CF composites (×2500).

Figure 2 shows the cryo-fractured surfaces of the matrix and composites. The surface of the PA11/PLA blend has a rough surface that contains the PLA phase in the PA11 phase. There are no obvious separated phase morphology or cavities that indicate self-compatibility between PA11 and PLA. The images of the PA11/PLA/CF composites show CF surfaces covered with the matrix. Although there are some voids and pullouts from the matrix in the 10 and 20 CF composites, it is seen that all of the fibers are completely embedded in the matrix. This demonstrates the matrix and polyamide-coated CF’s strong interfacial adhesion.

### 3.2 Thermal and Flammability Properties

**Figure 3:**
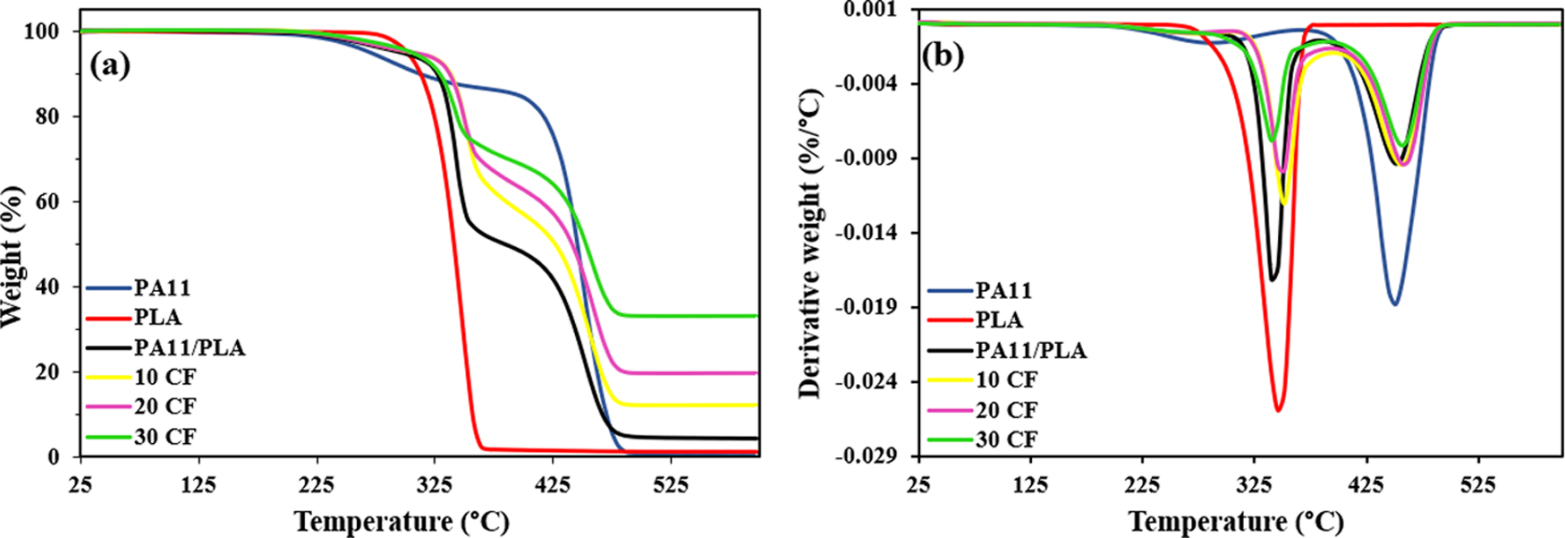
(a) TGA and (b) DTG results of samples.

Thermal stability is the capacity of a material to tolerate heat at a certain temperature without losing its physical characteristics, such as strength, hardness, and elasticity. During its service life, an EMI shield may heat up when exposed to electromagnetic waves, depending on the area of use. Therefore, thermal stability is a critical parameter for EMI applications and for melt processing of polymeric materials.^5,16^ So, the thermal stabilities of PA11/PLA/CF samples were investigated by TGA. The TGA and derivative thermogravimetry (DTG) curves are presented in Figure 3a,b. Also, the thermal degradation data of the samples are summarized in Table 1.

**Table 1:** Thermal Degradation Data of the Samples and Chopped CF

Sample	*T*_d5_ (°C)	*T*_d50_ (°C)	*T*_max-1_ (°C)	*T*_max-2_ (°C)	*T*_max-3_ (°C)	Residue (wt %, at 600 °C)
PA11	270.7	445.0	278.7	449.8		0.8
PLA	301.4	340.9	347.1			1.2
PA11/PLA	294.7	380.8	257.7	342.9	451.9	4.3
CF				420.9		98.1
10 CF	305.0	425.9	261.1	351.3	456.9	12.2
20 CF	305.2	440.6	262.2	349.2	458.2	21.8
30 CF	308.9	452.4	256.9	341.2	458.6	33.1

According to Figure 3, PA11 exhibited a two-step decomposition curve, the first attributable to the plasticizer in its structure and the other to the major decomposition of the PA. While the first step of degradation took place with a weight loss of about 7% in the range of 245–320 °C, the second step of degradation started at around 426 °C. Levchik et al. reported that mainly lactams, nitriles, and unsaturated hydrocarbons were formed during the main degradation of PA11. In the meantime, it was determined that gaseous species containing C=O, N–H, and CH_2_ groups were produced. The residue formed as a result of thermal degradation of PA11 was found to be similar to the original polymer with C–N and C=C bonds.^39,40^ The results demonstrated that PA11 has good thermal stability like PA6 and PA66. On the other hand, neat PLA showed a single-step decomposition starting at 300 °C. Thermal decomposition of PLA involves random main-chain scissions and unzipping depolymerization reactions.^41^ Three-step TGA curves were obtained showing the decomposition of each polymer. When the results were examined, it was observed that the first step of degradation of PA11 shifted to higher temperatures by blending PLA. When considering the degradation temperature of PLA, it was shown that the thermal stability of PLA improved as the amount of PA11 in the blend increased. Also, residual weight increased with blending. This thermal stability improvement is probably due to the “labyrinth effect” of the PA11 chains, which acts as an insulating barrier that can inhibit the evaporation process of PLA.^42^

The degradation behavior of CF was also investigated with the same test method applied to polymers. Table 1 shows that CF had a weight loss of 2% starting at 420 °C. This can be assigned to the degradation of the polyamide coating on the surface of CF. It can be observed in Figure 3 and Table 1 that CF effectively improved the thermal stability of the PA11/PLA blend. *T*_d5_, *T*_d50_, and *T*_max_ values of the PA11/PLA blend increased with increase of CF load, resulting in the addition of more thermally stable material as filler. As can be seen in Table 1, the residual weight of PA11/PLA was measured as 4.3% at 600 °C, which remarkably increased with the increasing CF amount. Since CF inhibits the diffusion of products that are decomposing and slows down the diffusion process, there has been an improvement in thermal stability. In addition, the barrier effect in the matrix increases with the increasing load of CF and thus the thermal decomposition temperature gradually rises.^5,43^

Flame resistance and self-extinguishing behavior as well as high thermal stability are expected from EMI shielding materials. Therefore, the flammability characteristics of PA11/PLA/CF composites were investigated by UL-94 and LOI, and the obtained data are summarized in Table 2. The PA11/PLA blend has an LOI value of 24.3% and UL-94 classification of V-2. During the vertical UL-94 test, after the combustion process started, dripping occurred and the cotton spread on the floor caught fire. With the addition of CF to the matrix, all of the samples were burned to the clamp in the first ignition. While the V classification did not change, the LOI increased with the increasing amount of CF.

**Table 2:** UL-94 Classification and LOI Values of PA11/PLA and Its Composites

	UL 94				
sample	first burning time (s)	second burning time (s)	dripping	V classification	LOI (%)
PA11/PLA	15.0	50	yes	V-2	24.3
10 CF	76.6	-	yes	V-2	25.3
20 CF	102.2	-	yes	V-2	26.2
30 CF	130.4	-	yes	V-2	27.1

### 3.3 Electrical Characteristics

**Figure 4:**
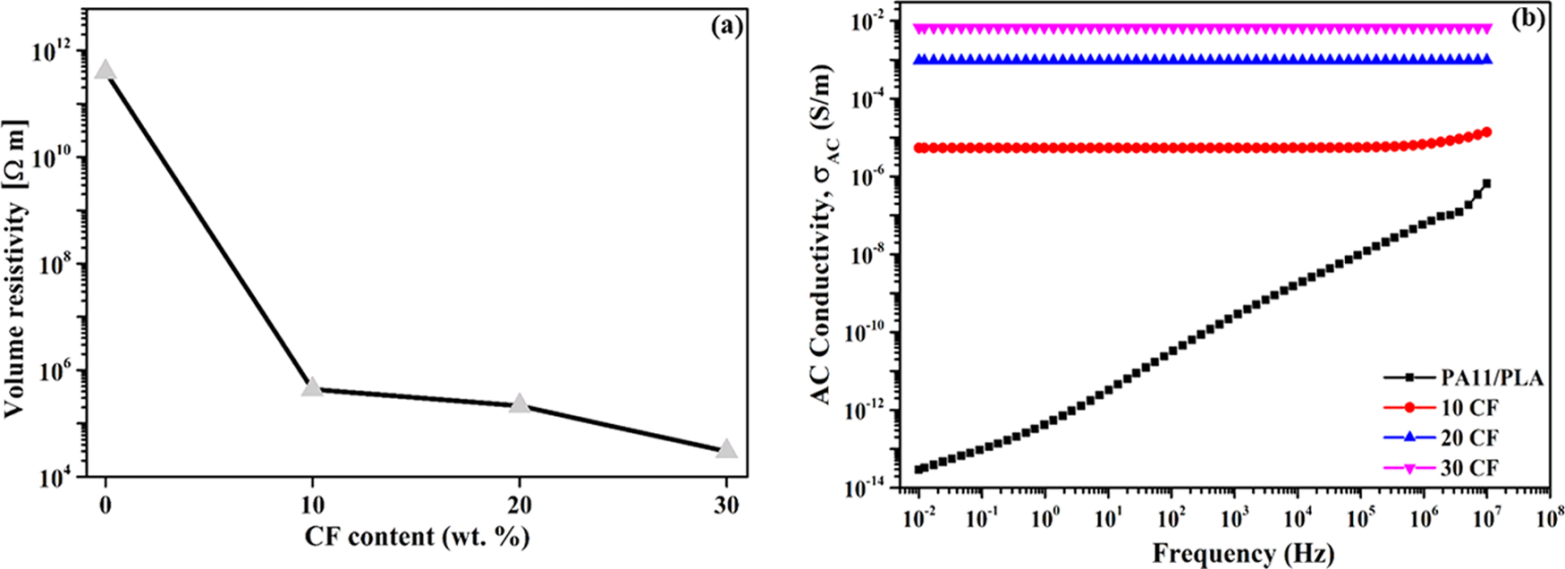
(a) Electrical volume resistivity and (b) AC conductivity of PA11/PLA/CF composites.

Figure 4a shows the DC electrical volume resistivity of PA11/PLA/CF as a function of the CF amount. The volume resistivity of the unreinforced PA11/PLA blend was measured as 3.95 × 10^11^ Ω·m. This result shows that the PA11/PLA blend is a typical electrically insulating material. As expected, the volume resistivity of the matrix decreased with the increase of the CF content. The volume resistivity of the matrix showed a sharp reduction with 10 wt % CF loading (4.38 × 10^5^ Ω·m). The reduction in resistivity was 6 orders of magnitude. This reduction can be explained by the percolation theory. The fibers behave as conductive islands in the electrically insulating polymer matrix at lower CF loading. With increase of CF loading, the possibility of conductive fibers coming into contact with one another in the matrix increases. At the percolation threshold, the fibers are in contact and form some conduction paths. Thus, electric charges or electrons can flow in the polymer matrix via the tunneling/hopping mechanism. The volume resistivity declines dramatically at this stage, and higher CF loadings do not cause any significant change.^44^ The percolation region for the PA11/PLA/CF composite is defined as the 0–10 wt % CF loading range. The volume resistivity remained almost in the same order of magnitude when the CF content was increased to 20 wt % (2.14 × 10^5^ Ω·m). The volume resistivity of 30 CF composites was 2.98 × 10^4^ Ω·m. As can be seen, there were no sharp changes in resistivity at high CF content. It can be said that the 30 CF composite contains many CF conductive networks and exhibits a semiconducting behavior.^45^ DC conductivities of PP/CF composites containing 5–20 wt % CF prepared by Kaushal and Singh were in the range of 10^–11^ to 10^–5^ S/m (10^11^ to 10^5^ Ω·m).^17^ The volume resistivity of a 15 wt % stainless-steel-fiber-reinforced PC/ABS composite, commercially named LNP FARADEX, developed by SABIC for use in EMI shielding applications, is in the range of 10^4^ to 10^6^ Ω·m.^46^ As can be seen, the volume resistivity of PA11/PLA/CF composites was determined to be at the same level as those in the literature and commercially used products.

Figure 4b shows a plot of the AC conductivities (σ_AC_) of the samples over a frequency range of 10^–2^ to 10^7^ Hz. It can be noted that the PA11/PLA blend exhibits a typical insulator behavior, including high-frequency dependency and low conductivity in accordance with volume resistivity values. The σ_AC_ of the PA11/PLA blend increased from 2.9 × 10^–12^ S/m at 0.01 Hz to 6.6 × 10^–5^ S/m at 10 MHz. As expected, CF improved the σ_AC_ of the matrix markedly. As can be seen, the conductivity varies with the amount of CF and the conductivity has gradually risen since the inclusion of 10 wt % CF. Conductivity occurs in polymeric composites via two basic mechanisms. One of them is the tunneling or hopping mechanism, in which electrons are transmitted through tunnels between noncontact particles. The amount of electron transport or current flow that can occur in this manner is constrained. This behavior is usually typical of insulating materials, such as the PA11/PLA blend, and electron transport, i.e., conductivity, increases with frequency.^5,47,48^ At low frequencies, the 10 CF composite demonstrated frequency independence, whereas at higher frequencies, it exhibited frequency dependence. Given that there are fewer free charge carriers in the low-frequency region, electrons move slowly in an electric field. Higher frequencies cause electrons to be compelled to migrate in the direction of the electric field, thus, improving conductivity.^48^ The other method of electron transmission is the mechanism in which the fibers in the matrix come into contact with each other and create conductive paths.^47,48^ The increase in conductivity and high conductivity values observed in 20 and 30 CF composites show that conductive paths occur in the composite structure. Also, these composites exhibited frequency-independent behavior. In other words, there is a conductive network formation in the entire measured area and this conductive network is stable.^48^ The conductivities of the 20 and 30 CF composites at 0.01 Hz are 0.095 and 0.68 S/m, respectively, and these values are at the same level as polystyrene composites containing 6–10% graphene prepared by solution mixing and hot pressing.^49^ Consequently, the PA11/PLA matrix reached the conductivity level by addition of 20–30 wt % CF. This conductive structure, which is made up of high aspect ratio fibers in contact with one another, is anticipated to have effective EMI shielding.

### 3.4 Dielectric Properties

**Figure 5:**
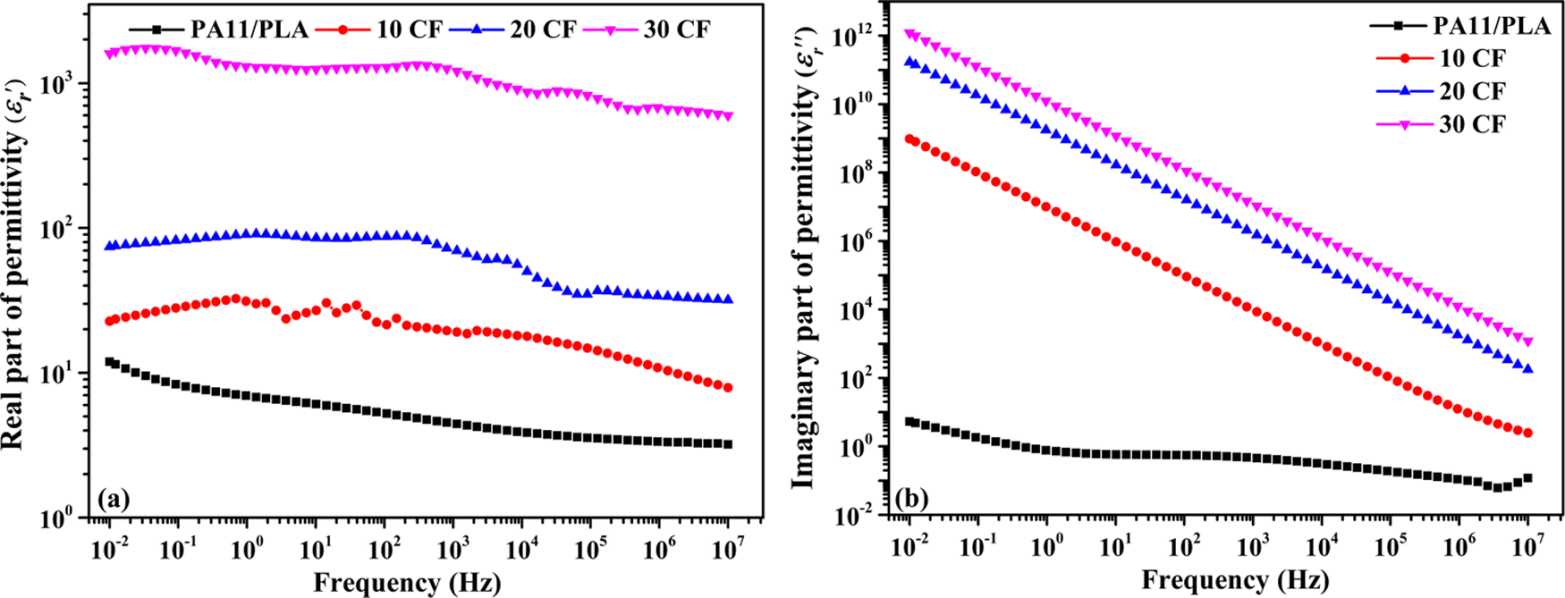
Variation of (a) real part and (b) imaginary part of dielectric permittivity.

The dielectric behaviors of the PA11/PLA/CF composite were also investigated. Complex relative dielectric permittivity (ε_r_*) is used to define how a material reacts to changing electric fields. It consists of two parts, real (ε_r_′) and imaginary (ε_r_″), which are important to interpret the EMI shielding performances of the composites.^45,48,50^ The dielectric constant, or ε_r_′, is a measurement of a material’s capacity to store energy from incident EM waves. It is generally connected to polarization, which is the separation and alignment of electric dipoles in a material caused by an electric field. Additionally, micro-capacitor-like structures that can develop in conductive polymer composites as a result of the filler and matrix serving as the electrode and dielectric insulator material have an impact on ε_r_′.^27,45,51^ The quantity of energy dissipated in the material exposed to the electric or electromagnetic field is indicated by the imaginary component of the complex dielectric permittivity or dielectric loss (ε_r_″). It primarily has to do with conductivity, ε″ = σ/2π*f*.^45,51^ The ε_r_′ and ε_r_″ of the samples were measured at room temperature in a frequency range of 10^–2^ to 10^7^ Hz and graphed in Figure 5a,b.

As can be seen in Figure 5, ε_r_′ and ε_r_″ exhibited frequency-dependent behavior. Both ε_r_′ and ε_r_″ decreased with increasing frequency. This behavior has been reported to be the same for almost all carbon-doped composites.^17,45,47,52−54^ The downward tendency of ε_r_′ and ε_r_″ can be assigned to the variation of polarization behavior with frequency. Polarization usually takes place with dipole, electronic, and ionic contributions. All of these contributions have an effect on dielectric permittivity at low frequencies. As the frequency of the incident EM wave increases, the dipoles are unable to keep up with the electromagnetic field’s quickly changing state. Eventually, the dipoles start to lag and a mismatch occurs between dipolar polarization and electrical polarization. Thus, the polarization effect and consequently the dielectric characteristics are reduced.^47,50,54,55^ On the other hand, it was found that ε_r_″ was higher than ε_r_′ for all frequency ranges. The higher ε_r_″ can be attributed to dipole polarization caused by defects in the CF structure and functional groups, and to conductive losses due to increased electrical conductivity.^26,56^

Considering the effect of CF amounts on dielectric properties, it is seen that both ε_r_′ and ε_r_″ increase with increasing CF amount in the whole frequency range. On the other hand, the actual increase is more pronounced at low frequencies. The Maxwell–Wagner–Sillars (MWS) effect can explain this rising trend.^45,53^ The filler–matrix interface alters the dielectric characteristics, according to the MWS effect. Accordingly, when a current passes through the interface of two materials with different relaxation times, charges can collect at the interface. As the amount of CF in the matrix increases, more interfaces will be formed, so more interfacial polarization will occur and thus ε_r_′ and ε_r_″ will increase.^17,53,57^ Besides, the formation of a micro-capacitance structure also causes an increase in dielectric permittivity. The fibers in the matrix act as microelectrodes and the matrix acts as a dielectric, forming a micro-capacitor in composites. With the increase in the amount of CF added to the matrix, there was an increase in the number of micro-capacitors and thus the ε_r_′ and ε_r_″.^54,55^ These findings are consistent with those of polymer/CF composites in the literature.^5,17,53,54^ The enhanced dielectric characteristics are anticipated to improve the material’s EMI shielding capability.

### 3.5 EMI Shielding Effectiveness

**Figure 6:**
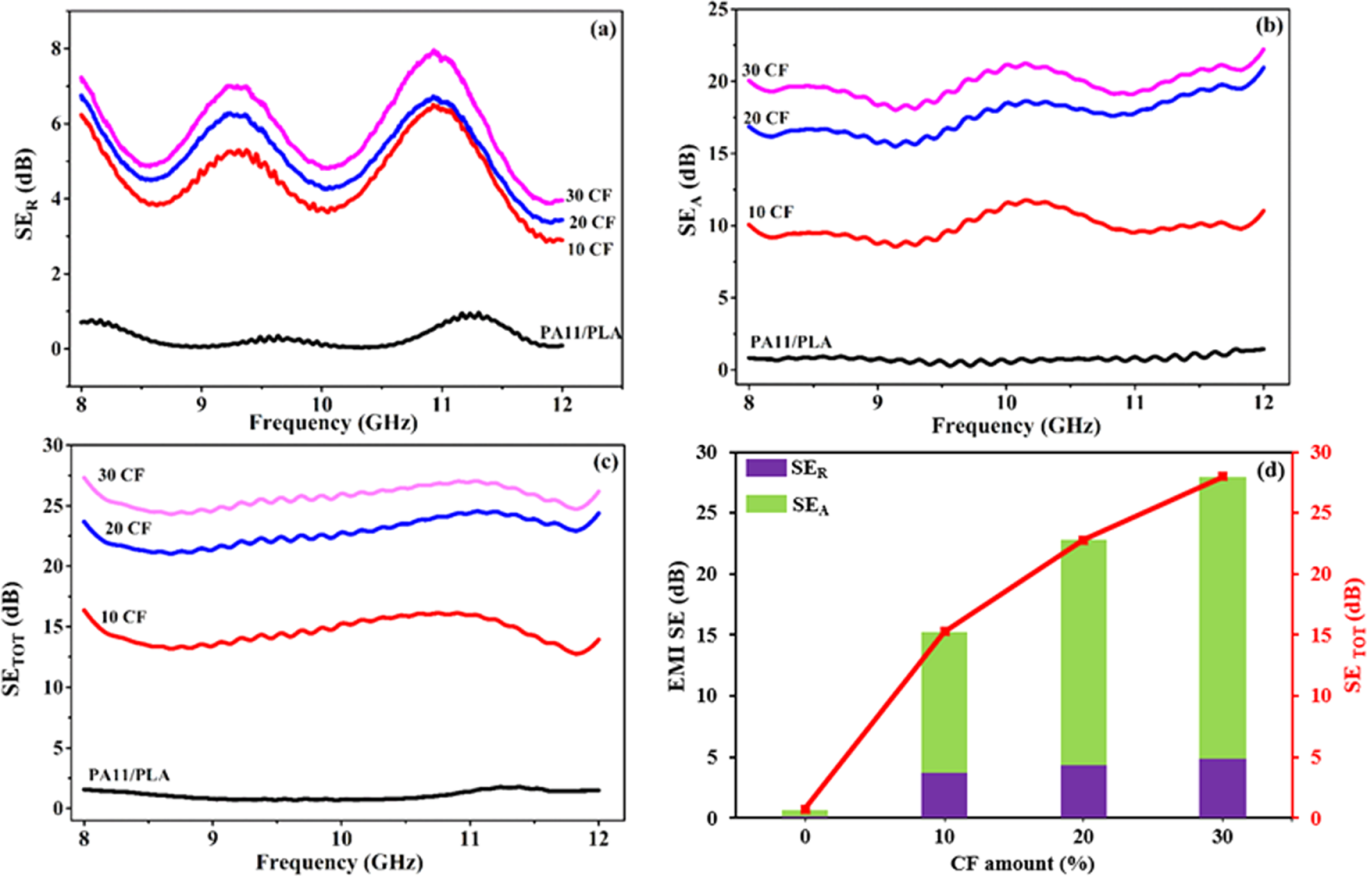
Variation of (a) SE_R_, (b) SE_A_, and (c) SE_TOT_ (EMI SE) of PA11/PLA/CF composites with frequency in X-band, and (d) comparison of SE_R_, SE_A_, and SE_TOT_ as a function of the CF amount at 10 GHz.

As previously mentioned, EMI shielding comprises two fundamental mechanisms, i.e., reflection (SE_R_) and absorption (SE_A_). The sum of these gives the EMI shielding effectiveness (SE_TOT_) of the material. Figure 6a–d shows the EMI shielding capabilities of the PA11/PLA/CF composites as well as the contribution of SE_R_ and SE_A_ to the SE_TOT_. The SE_TOT_ values of the samples are nearly frequency independent, as can be seen in Figure 6c. The PA11/PLA matrix shows 0–2 dB SE_TOT_ throughout the whole frequency range. Because of its low conductivity and dielectric permittivity, the polymeric matrix is transparent to EM waves, which is generally observed and expected.^23,47,51^ The SE_TOT_ of the matrix increased remarkably with the increasing amount of CF. As shown in Figure 6d, the SE_TOT_ value of the matrix is 0.7 dB at 10 GHz and increases to 15, 23, and 28 dB, respectively, when 10–20–30 wt % CF is added. Shielding requirements differ depending on the application. It is generally accepted that EMI SE values of 20 dB and higher are sufficient for practical applications. For instance, the shielding requirement for desktop and laptop computers is 15–20 dB. An EMI SE value of 20 dB means that 99% intensity of incident EM fields can be shielded. When the EMI SE value reaches 30 dB, it can be said that 99.9% of the intensity of EM fields can be shielded.^22,24,30,53,54,58^ An acceptable level of EMI SE could be achieved by adding 20–30 wt % CF. The rise in conductivity, which is also depicted in Figure 4, the formation of conductive paths within the structure, and the enhancement of the dielectric characteristics are all responsible for the increase. The incident EM waves are reflected or absorbed by the conductive CF network. At low CF amounts, gaps in the network cause wave transitions, whereas with increase of CF, a denser network forms, and wave transitions become increasingly difficult.^24,59^ The resulting increase is consistent with data in the literature.^17,21,24,27,30,53,59^ The effects of CF addition (0–10 vol %) on the EMI SE value of the PP composite produced in solid and foam forms were examined in the study by Ameli et al.^53^ EMI SE values of 3.2 mm thick samples in solid form were measured in the X-band and the maximum EMI SE value was acquired as 16.3 dB at 10 vol % CF loading. It was revealed in another study that 2 mm thick PP/CF composites in the X-band attained EMI SE of 24, 30, and 32 dB at 10–15–20 wt % CF content.^17^ As can be seen, the EMI SE values of PA11/PLA/CF composites are competitive with those of other CF-reinforced composites. Furthermore, the sample thicknesses in the comparative studies are higher than those in our study. Despite being thinner, the PA11/PLA/CF samples exhibit higher EMI SE values. The thickness of the shield material also affects the EMI SE. The conductive network structure in the thick material can further attenuate the incident EM wave. It is well known that when sample thickness increases, the EMI SE value rises.^16,60^ According to Luo et al., as the thickness of the MXene/natural rubber nanocomposite increased from 34 to 248 μm, the EMI SE value at 12.4 GHz increased from 12.2 to 29.4 dB.

Contributions of reflection and absorption mechanisms to EMI SE for detailed evaluation are shown in Figure 6a,b. The main cause of EMI shielding through reflection is an impedance mismatch between the shielding material’s surface and free space. Furthermore, the shield must involve mobile charge carriers like electrons and holes that can interact with the incoming EM wave and produce a counter field known as an induced field or a scattering field for reflection to be realized. Metals are common materials that provide shielding by reflection due to their high conductivity.^4,27^ Since the impedance of the conductive shield is lower than the impedance of free space, a greater impedance mismatch causes significant EM wave reflection.^4^ To absorb the nonreflective portion of an incoming EM wave, the shield must include magnetic and electric dipoles which can interact with the EM wave’s magnetic and electric fields. Thus, when the EM wave enters the shield, it loses its energy due to dielectric (polarization) and ohmic losses. Absorption is a function of conductivity and permittivity. Conductive networks of a structure increase ohmic losses while materials with high dielectric constant values provide dipoles.^4,27^

In Figure 6a, low SE_R_ values between 3 and 8 dB indicate the impedance matching between composites and free space. The value of SE_R_ increased with the increase of the CF amount. With increased CF amount, the conductivity of the matrix increased, causing an impedance mismatch between the shield and the free space and enhancing the contribution of reflection.^58,61^ On the other hand, the SE_R_ curves showed small fluctuations in the X-band frequency. In the composites, when incident EM waves interact with conductive fibers acting as resonators, they can induce electric currents at certain frequencies. If the conductive fibers are aligned in a particular orientation within the matrix, they can resonate at a particular frequency, causing a peak in the SE_R_ curve at that frequency.^25^Figure 6a,b demonstrates that the SE_A_ value always exceeds the SE_R_ value in all CF amounts. The fact that SE_A_ is higher than SE_R_ indicates that the overall EMI shielding performance of PA11/PLA/CF composites is primarily accomplished through absorption rather than reflection.^53,61^Figure 6d depicts a more illustrative graph of the individual contributions to SE_T_ at a constant frequency of 10 GHz. The SE_A_ value of all composites dominates by a significant margin, making up about 82% of the SE_T_. The assumption that the absorption mechanism is dominant, on the other hand, is not totally accurate. Due to the impedance mismatch, the incident EM wave first strikes the surface of the shield and reflects. The nonreflective portion of the electromagnetic wave keeps moving toward the shield. High SE_R_ values of more than 3 dB caused by the wave striking the shield surface indicate that more than half of the incoming EM waves are reflected before penetrating the shield material.^58,61^ This implies that reflection is dominant.

**Figure 7:**
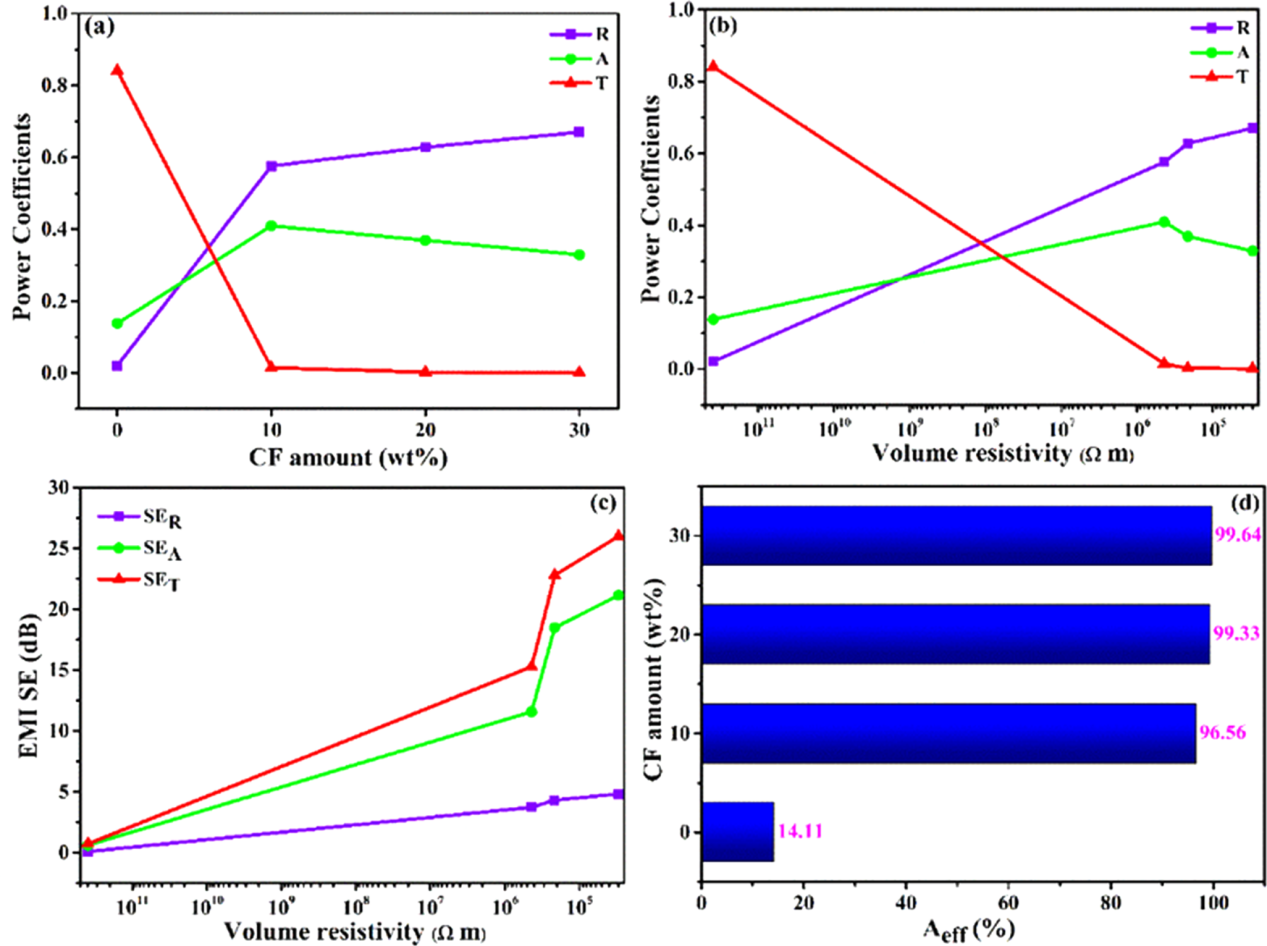
Power coefficients as a function of (a) the CF amount and (b) volume resistivity. (c) EMI SE as a function of volume resistivity. (d) Effective absorbance.

Figure 7a shows the power coefficients (*A*, *R*, and *T*) as a function of the CF amount at 10 GHz computed from the scattering parameters in accordance with eq 2 to assess the EMI shielding mechanism more clearly. The PA11/PLA matrix exhibits a high *T* value of 0.84, showing that it is transparent to EM waves. The *T* value dropped drastically as CF was added and its amount was raised, while *R* and *A* increased. This could be because of the addition of CF, which increases electrical conductivity and dielectric losses. *R* continued to increase at high CF amounts while *A* decreased, because the impedance mismatch between the free space and the shield surface will grow as conductivity increases.^47,58,61^Figure 7b illustrates the connection between the volume resistivity and the power coefficients of the composites. *R* increased with the reduction in volume resistivity, that is, with the increase in conductivity.

On the other hand, *A* increased first and then reduced slightly as the conductivity increased. This confirms that as the CF amount and conductivity level rise, the impedance mismatch intensifies. The correlation between volume resistivity and SE values is shown in Figure 7c. As can be seen, the reflection power coefficient is higher than the absorption, while the SE_R_ value is quite low compared to SE_A_. This inverse relationship can be explained by the differences in power coefficients and shielding efficiency notions. *R*, A, and *T* are quantitative notions of the power balance of the incoming and attenuated EM waves, but SE is a relative notion that does not directly depend on the absolute power.^61^ SE_A_ is the ability to disperse the EM wave that can enter the shield after reflection, while *A* represents the ratio of the power attenuated by the shield to the total incoming power. That is, the contribution of absorption to total shielding depends on the material’s capacity to attenuate the nonreflected EM wave and the thickness of the shield material.^47,58^ In other words, PA11/PLA/CF composites have EM wave absorption capabilities as well, although the shielding occurs primarily through reflection.

The ratio of the incoming power to the absorbed power is defined as effective absorbance (*A*_eff_). The *A*_eff_ values were computed using eq 5 and are shown in Figure 7d as a function of the amount of CF to more accurately assess the absorption potentials of the composites. The *A*_eff_ value of the matrix was 14%, and it increased to 96% with the addition of 10 CF and eventually reached 99% as the amount of CF increased. This indicates that CF considerably improves the matrix’s capacity to absorb EM waves, and this phenomenon is due to increasing polarization and ohmic losses after CF addition.

## 4 Conclusions

In summary, CF-reinforced environmentally friendly PA11/PLA composites were successfully manufactured as EMI shielding material. The composite samples were fabricated by commercial twin-screw extrusion and injection/compression molding methods. The effects of CF amounts on thermal stability, flammability, volume resistivity, AC conductivity, and dielectric and EMI SE performances were examined. According to SEM images, there is a strong interfacial adhesion between the PA11/PLA matrix and CFs. TGA showed that CF improves the thermal stability of the matrix by preventing the diffusion of degrading products and decreasing the diffusion rate. Flammability tests showed that the LOI value increased with the addition of CF, while the UL-94 classification remained V-2. Still, the 30 CF composite exhibited a good LOI of 27%. The volume resistivity of the matrix, which is 3.95 × 10^11^ Ω·m, decreased to 10^5^ and 10^4^ Ω·m, respectively, by adding 20–30 wt % CF. The obtained volume resistivity values are at the same level with the literature data and commercial products. Moreover, the AC conductivities of the composites were evaluated in the range of 10^–2^ Hz to 10^7^ Hz, and it was observed that the conductivity level reached 0.095 and 0.68 S/m, respectively, in the composites containing 20–30 wt % CF. Based on this, it was concluded that a conductive network structure was created in the matrix. Besides AC conductivity, the real and imaginary parts of dielectric permittivity of PA11/PLA/CF composites increased by several orders of magnitude with CF loading. It was found that ε_r_″ was higher than ε_r_′. Composites that benefit from the conductive network structure and high dielectric properties exhibited acceptable levels of EMI SE in the X-band frequency. While the matrix was transparent to EM waves, composites containing 10–20–30 wt % CF exhibited EMI SE of 15, 23, and 28 dB, respectively, at 10 GHz. It was observed that the obtained EMI SE values were comparable to the polymer/carbon fiber composites available in the literature and even higher than the thicker composite samples. In the calculations to determine the EMI shielding mechanism, reflection was found to be the dominant mechanism, similar to the literature data. Finally, in this study, as an EMI shield, CF-reinforced bio-based polymeric composites were shown to be promising. The required 20 dB EMI SE value for practical applications has been achieved and an alternative shielding material to metals has been presented for electronic equipment such as computers and radio frequency circuits.
